# First Line of Defense: Innate Cell-Mediated Control of Pulmonary Aspergillosis

**DOI:** 10.3389/fmicb.2016.00272

**Published:** 2016-03-03

**Authors:** Vanessa Espinosa, Amariliz Rivera

**Affiliations:** ^1^Center for Immunity and Inflammation, New Jersey Medical School, Rutgers-The State University of New JerseyNewark, NJ, USA; ^2^Graduate School of Biomedical Sciences, New Jersey Medical School, Rutgers-The State University of New JerseyNewark, NJ, USA; ^3^Department of Pediatrics, New Jersey Medical School, Rutgers-The State University of New JerseyNewark, NJ, USA

**Keywords:** aspergillosis, innate cells, monocytes subsets, mechanisms of resistance, neutrophils, dendritic cells (DC)

## Abstract

Mycotic infections and their effect on the human condition have been widely overlooked and poorly surveilled by many health organizations even though mortality rates have increased in recent years. The increased usage of immunosuppressive and myeloablative therapies for the treatment of malignant as well as non-malignant diseases has contributed significantly to the increased incidence of fungal infections. Invasive fungal infections have been found to be responsible for at least 1.5 million deaths worldwide. About 90% of these deaths can be attributed to *Cryptococcus, Candida, Aspergillus*, and *Pneumocystis*. A better understanding of how the host immune system contains fungal infection is likely to facilitate the development of much needed novel antifungal therapies. Innate cells are responsible for the rapid recognition and containment of fungal infections and have been found to play essential roles in defense against multiple fungal pathogens. In this review we summarize our current understanding of host-fungi interactions with a focus on mechanisms of innate cell-mediated recognition and control of pulmonary aspergillosis.

## Introduction

Immunocompromised individuals comprise a growing population in today's world. In part, this is due to the increased use of immunosuppressive drugs as therapies for diverse disease states. Thus, a variety of patients are increasingly more susceptible to develop invasive fungal infections. *Aspergillus fumigatus* is the etiological agent of over 90% of the invasive aspergillosis (IA) cases and it is considered the most common inhaled fungal pathogen (Dixon et al., 1996; Hohl and Feldmesser, 2007; Lehrnbecher et al., 2010). Even with diagnosis and treatment, individuals suffering from IA rarely recover. Exposure to *A. fumigatus* spores is a daily event, and for most individuals exposure to this environmental fungus is without consequence (Ben-Ami et al., 2010). Immune responses to *A. fumigatus* are central in preventing IA, and are likely responsible for the absence of disease manifestations in people with an intact immune system. Several recent reviews have detailed the important contributions of adaptive immunity to antifungal defense (Wuthrich et al., 2012a; Rivera, 2014; Verma et al., 2015). In this review we will focus our discussion on the recognition of the pathogen, the role of the innate immune system in response to respiratory fungal infection, and how diverse innate cell populations orchestrate antifungal defense against *A. fumigatus*.

## *Aspergillus fumigatus* and related diseases

*Aspergillus fumigatus* is regarded as one of the most prevalent airborne fungal pathogens capable of causing severe to fatal invasive infections in immunocompromised individuals (Dixon et al., 1996; Hohl and Feldmesser, 2007; Lehrnbecher et al., 2010). Once inhaled, the conidia of *A. fumigatus* are small enough (2–3 microns) to enter the terminal respiratory airways, and reach the pulmonary alveoli (Ben-Ami et al., 2010). It is estimated that humans inhale several conidia per day, which are efficiently cleared by the pulmonary innate immune system (Margalit and Kavanagh, 2015). If not, they will germinate into hyphal structures, which can damage lung tissue (Dagenais and Keller, 2009). The innate immune system is the first line of defense against metabolically active and swelling conidia. Important innate cells in defense against aspergillosis include macrophages, neutrophils, monocytes and dendritic cells (Margalit and Kavanagh, 2015) (Table 1).

**Table 1:** **Summary of innate cell defense in *A. fumigatus* infection**.

**Cell type**	**Contribution to defense**
Epithelial cells	Antimicrobial peptides such as lactoferrin, chitinase, and β-defensins Production of pro-inflammatory cytokines (TNFα, GM-CSF, IL-8, and the β-defensins HBD2 and HBD9)
Alveolar macrophages	ROS and phagosomal acidification Cytokine and chemokine production including neutrophil attractants CXCL1 and CXCL2
Neutrophils	ROS generation via NADPH oxidase, lactoferrin production, NETosis, and through the release of antimicrobial proteases by degranulation
Eosinophils	Antimicrobial proteins present in their granules
Mast cells	Degranulate and release a variety of enzymes and bioactive substances, such as histamine and tryptase, that mediate pulmonary inflammation and airway constriction
Platelets	Damage the fungal cell wall upon exposure through the release microbicidal proteins stored in their granules
Natural killer cells	Release of perforins and cytokine production such as IFNγ
Conventional DCs	Function as an important regulator of the inflammatory response via IL-2 production
Plasmacytoid DCs	Type I IFN production, pET formation, and release of antifungal effector molecules such as zinc chelators like calprotectin and iron-binding proteins like lactoferrin
CCR2^+^ inflammatory monocytes	Differentiate into inflammatory macrophages or into TIP DCs, which are capable of internalization and elimination of conidia Shape the adaptive immune response toward Th1 instead of Th17

One of the most deleterious complications that can affect an immunocompromised individual is invasive aspergillosis (IA; Hohl and Feldmesser, 2007). Examples of susceptible immunocompromised patients include: those who are undergoing chemotherapy for acute leukemia, recipients of allogeneic haematopoietic stem cell transplants as well as solid-organ transplants, those under corticosteroid treatment for graft-vs.-host disease (GVHD), patients with aplastic anemias and prolonged neutropenia, patients that suffer from neutrophil defects such as chronic granulomatous disease (CGD), and patients suffering from advanced human immunodeficiency virus disease (HIV; Ben-Ami et al., 2010). Infection occurs primarily in the lungs of the patients, but dissemination to practically every organ can occur in the most severe of cases (Segal, 2009).

Some of the most prominent characteristics of IA include: filamentous growth in the pulmonary parenchyma, angioinvasion, intravascular thrombosis, tissue infarction, and haematogenous dissemination (Ben-Ami et al., 2010). Dissemination of aspergillosis to the central nervous system is a devastating effect of IA, which is characterized by the onset of seizures as well as other focal neurologic signs (Segal, 2009). IA has been found to be a leading cause of death among hematology patients (Latge, 1999). It is estimated to occur in 5–25% of acute leukemia patients, 5–10% after allogeneic bone marrow transplantation, and 0.5–5% after cytotoxic treatment of blood diseases as well as solid-organ transplantation (Latge, 1999). IA is also considered to be the main fungal infection found in cancer patients (Bodey et al., 1992; Wald et al., 1997; Kaiser et al., 1998; Lehrnbecher et al., 2010). The average incidences described are probably underestimations of the actual number of incidences since the diagnostic tests available are of low sensitivity (Bodey et al., 1992; Wald et al., 1997; Kaiser et al., 1998; Lehrnbecher et al., 2010; Brown et al., 2012).

*A. fumigatus* has also been shown to cause other diseases such as allergic bronchopulmonaryaspergillosis (ABPA) and aspergillomas (Latge, 1999). ABPA is the most severe allergic complication, (Latge, 1999) and it usually occurs in patients suffering from atopic asthma (1–2% develop ABPA) or cystic fibrosis (7–35% develop ABPA; Knutsen and Slavin, 1992; Moss, 2002). The disease manifests itself as a bronchial asthma that has transient pulmonary infiltrates, which may lead to proximal bronchiectasis and lung fibrosis (Cockrill and Hales, 1999; Moss, 2005). In the most severe of cases, ABPA can lead to respiratory failure and the fatal destruction of the infected lung (Knutsen et al., 2002; Moss, 2002, 2005). Aspergilloma, on the other hand, has been shown to occur in the preexisting lung cavities that have been caused by various lung disorders such as tuberculosis and sarcoidosis (Kirsten et al., 1992; Fujimura et al., 1998). Aspergilloma is characterized by a spheroid mass of hyphae that is embedded within a proteinaceous matrix in the external lining of the cavity (Latge, 1999).

## Recognition of *Aspergillus fumigatus* by innate cell receptors

### C-type lectin receptors (Dectin-1 and Dectin-2)

Upon inhalation, conidia mature and begin to swell, which leads to the loss of their RodA hydrophobic layer exposing the β-glucans in their cell wall (Aimanianda et al., 2009). β-glucans are recognized by the C-Lectin receptor, Dectin-1 (Hohl et al., 2005; Werner et al., 2009). Dectin-1 is expressed on macrophages, neutrophils, and dendritic cells (Werner et al., 2009). *In vitro*, it has been shown that Dectin-1 dependent alveolar macrophage production of cytokine and chemokines does not depend on the phagocytosis of the conidia, but on its morphology (Luther et al., 2007). Dectin-1 has been shown to be activated only in the presence of swollen, but not resting conidia (Gersuk et al., 2006; Aimanianda et al., 2009). Dectin-1 signals through Syk kinase, leads to the activation of NFκB, and the production of tumor necrosis factor (TNFα), IL-10, IL-6, IL-1α, granulocyte macrophage colony stimulating factor (GM-CSF), macrophage inflammatory protein α and β (MIP-1α, and MIP-1β; Hohl et al., 2005; Steele et al., 2005; Faro-Trindade et al., 2012). Dectin-1 has also been shown to have an important role in neutrophil recruitment (Werner et al., 2009). In Dectin-1 deficient mice, defects in neutrophil recruitment were due to unresponsive alveolar macrophages that were unable to produce chemoattractants (Werner et al., 2009).

Dectin-2, in contrast, recognizes α-mannans, which are found in the outer layer of the cell wall (Levitz, 2010; Sun et al., 2014). Dectin-2 has been shown to be mainly expressed on macrophages as well as dendritic cells (Sun et al., 2014). Detection of swollen conidia leads to the production of IL-1β, IL-10, IL-23p19, and TNFα via NFκB mediated by Syk (Sun et al., 2013, 2014). Blocking of Dectin-2 and Syk results in reduced conidial killing in macrophages differentiated from a human monocytic cell line (Sun et al., 2014).

### Toll-like receptors (TLRs)

Toll-like receptors (TLRs) are membrane receptors that have a leucine-rich extracellular domain that recognizes pathogen-associated molecular patterns (PAMPs) as well as an intracellular Toll/interleukin-1 receptor (TIR) domain needed for downstream signaling (Kawai and Akira, 2007). Once TLRs recognize the pathogen, the signaling cascade leads to the activation of NFκB and other transcription factors, which leads to cytokine and chemokine production (Kawai and Akira, 2007).

TLRs have been found to play important roles in recognition of *A. fumigatus* for host defense although there is conflicting data, which could be attributed to differences in experimental design (Steele et al., 2005). They are primarily expressed on the cell surface of monocytes, macrophages, and dendritic cells (Takeda and Akira, 2005). TLR1^−∕−^ murine bone marrow-derived macrophages had reduced amounts of IL-6, TNFα, CXCL2, and IL-12p40 in response to *A. fumigatus* conidia (Rubino et al., 2012). Cytokine production was diminished in TLR2^−∕−^, TLR4^−∕−^ and TLR6^−∕−^ macrophages, but not in TLR3^−∕−^ or wild-type macrophages. In terms of survival, TLR1^−∕−^, TLR2^−∕−^, TLR4^−∕−^, and TLR6^−∕−^ have been shown to be non-essential, and do not make immunocompetent mice more susceptible to *A. fumigatus* infection (Dubourdeau et al., 2006; Rubino et al., 2012). In another study performed *in vitro*, TLR2^−∕−^ murine alveolar macrophages (AMs) infected with *A. fumigatus* were found to have an impaired inflammatory response due to their deficiency in TNFα production (Steele et al., 2005). Because AMs were still able to produce some detectable TNFα, TLR2 was determined to be non-essential for TNFα production, but necessary for Dectin-1 mediated production of TNFα (Steele et al., 2005). When AMs were treated with Dectin-1 blocking antibody, there was an observed 80% decrease in cytokine production, which was consistent in both TLR2^−∕−^ and wild-type AMs (Steele et al., 2005). In addition, TLR2^−∕−^TLR4-/- mice, were found to have deficiencies in neutrophil recruitment compared to the single knockouts indicating that both receptors are required for an optimal immune response (Meier et al., 2003). TLR2 and TLR4 signaling requires the adaptor MyD88 adapter-like (Mal)/TIRAP in the myeloid differentiation primary response gene 88 (MyD88) dependent pathway (Horng et al., 2002). TLR4, on the other hand, can signal through another adaptor molecule, TIR-domain-containing adapter-inducing interferon-β (TRIF)-related adapter molecule (TRAM/TICAM-2), in the MyD88 independent pathway (Yamamoto et al., 2003).

In contrast to previous studies (Meier et al., 2003; Bellocchio et al., 2004; Rubino et al., 2012), other work has recently implicated a role for TLR3^−∕−^ in *A. fumigatus* infection. TLR3^−^/^−^ mice were observed to have deficiencies in dendritic cell (DC) migration to the lymph node, which affected their ability to prime T cells (Carvalho et al., 2012). Consistent with this finding, TLR3^−∕−^ mice lacked the ability to produce a CD8^+^ T cell response in response to *A. fumigatus* infection (Carvalho et al., 2012). TLR3 as well as TLR4 can signal through the adaptor protein TRIF (Kawai and Akira, 2007), and TRIF^−∕−^ mice displayed sustained inflammatory cell recruitment to the lungs in comparison to MyD88^−∕−^ and wild-type mice that were chemically immunocompromised to serve as models of IA (de Luca et al., 2010). In addition, TLR3-expressing lung epithelial cells were shown to activate indoleamine 2,3-dioxygenase, which is an interferon (IFN)- inducible enzyme that degrades the amino acid tryptophan and suppresses adaptive T cell immunity (de Luca et al., 2010).

TLR9 is expressed on a variety of cells such as macrophages and monocytes (Ramirez-Ortiz et al., 2008). During phagocytosis of *A. fumigatus*, TLR9 recognizes the exposed and unmethylated CpG DNA (Ramirez-Ortiz et al., 2008). TLR9^−∕−^ neutropenic mice exhibited a decreased inflammatory response compared to wild-type 2 days post infection, but was significantly increased 4 days post infection indicating an immunoregulatory role for TLR9 in *A. fumigatus* infection (Ramaprakash et al., 2009). Dectin-1 expression was also found to be decreased in TLR9^−∕−^ mice, which could explain why there is a delayed immune response since Dectin-1 is important for recognition of *A. fumigatus* swollen conidia (Ramaprakash et al., 2009).

### Myeloid differentiation primary response gene 88 (MyD88)

MyD88, the universal adapter through which all TLRs except TLR3 signal, has been shown to play an important role early in the inflammatory response against *A. fumigatus* (Ramaprakash et al., 2009). MyD88^−∕−^ mice were shown to have delayed fungal clearance for the first 2 days, but were comparable to wild-type mice at about 3 days (Bretz et al., 2008). Early on, MyD88^−∕−^ lungs appeared to have more necrotic tissue, and using a fluorescent *A. fumigatus* strain, a deficiency in macrophage uptake was observed (Bretz et al., 2008). Also, there was decreased cytokine production of interleukin (IL)-1β, IL-6, keratinocyte-derived chemokine (KC/CXCL1), IFNγ, but increased amounts of TNFα and MIP-1α in MyD88^−∕−^ mice (Bretz et al., 2008). The normalization observed at day three indicates that there are alternative pathways involved in fungal clearance that are MyD88 independent (Margalit and Kavanagh, 2015). Recent studies further suggest that MyD88 signaling in defense against IA is crucially active on lung epithelial cells and is required for optimal production of neutrophil-recruiting chemokines (Jhingran et al., 2015).

## Innate cell subsets and their roles in defense against aspergillosis

### Epithelial cells

**Figure 1:**
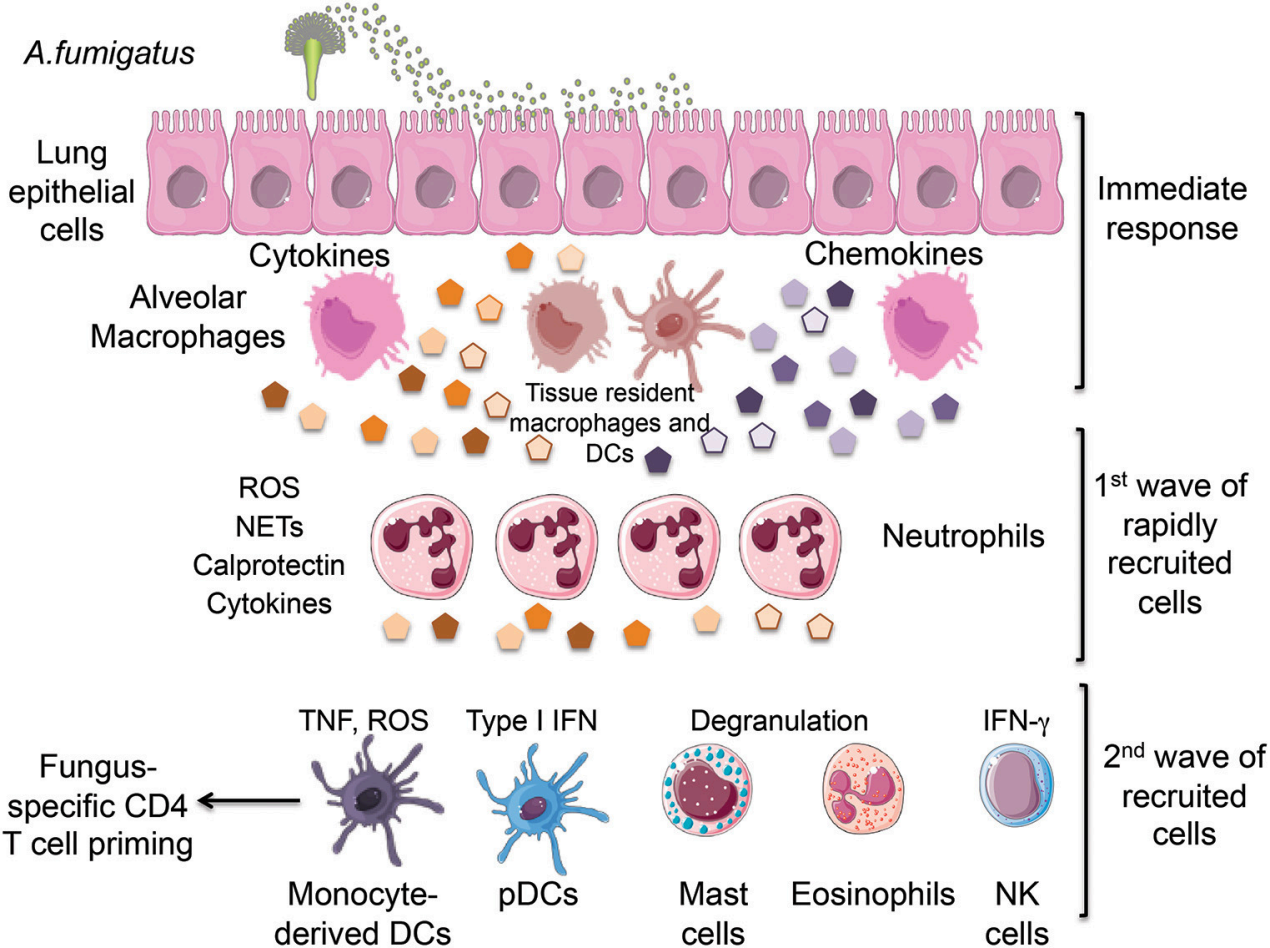
**Inhalation of *Aspergillus fumigatus* (*A. fumigatus*) conidia leads to the initial recognition of infection by lung epithelial cells and tissue-resident innate cells including alveolar macrophages and dendritic cells**. This immediate response results in the production of chemokines that promote the rapid recruitment of neutrophils followed by the subsequent arrival of monocytes, pDCs, mast cells, eosinophils and NK cells. All of these innate cells cooperate in the elimination of fungal conidia by producing a combination of cytokines and protective factors. ROS, reactive oxygen species; NETs, neutrophil extracellular traps; TNF, tumor necrosis factor; IFN, interferon; pDCs, plasmacytoid dendritic cells. We would like to thank Servier Medical Art (http://www.servier.com) for figure graphics of immune cells.

The airway epithelium is the first point of contact for fungal spores upon inhalation, which leads to the initiation of the innate immune response (Figure 1; Paris et al., 1997). The respiratory epithelium consists of a variety of cell types such as mucous-secreting goblet cells, ciliated cells, and most importantly, respiratory epithelial cells (Paris et al., 1997). Respiratory epithelial cells release a broad range of antimicrobial peptides such as lactoferrin, chitinase, and β-defensins (Alekseeva et al., 2009; Balloy and Chignard, 2009). The tracheobronchial epithelial cells, Type II alveolar epithelial cells, and endothelial cells have been shown to have the ability to internalize conidia, which are then trafficked to late endosomes for processing (Paris et al., 1997; Filler and Sheppard, 2006). In comparison to other phagocytosing cells such as macrophages and neutrophils, epithelial cells are less efficient in fungal elimination (Wasylnka and Moore, 2003). Respiratory epithelial cells also express recognition receptors such as CLRs and TLRs (Sun et al., 2012). Upon challenge of the human bronchial epithelial cell line with swollen *A. fumigatus* conidia, Dectin-1 expression was induced in a TLR2-dependent manner, which induced the expression of ROS as well as TNFα, GM-CSF, IL-8, and the β-defensins HBD2 and HBD9 (Balloy et al., 2008; Sun et al., 2012). Dectin-1 blockade inhibited the expression of these factors indicating that airway epithelial cells require Dectin-1 for the upregulation of pro-inflammatory cytokines as well as antimicrobial factors (Sun et al., 2012).

### Alveolar macrophages (AM)

Alveolar macrophages have been shown to uptake as well as kill conidia through two known mechanisms: Reactive oxygen species (ROS) generation and phagosomal acidification (Ibrahim-Granet et al., 2003; Philippe et al., 2003). ROS generation occurs in response to swollen but not resting conidia, which leads to the recruitment of cytosolic proteins (p47^phox^, p67^phox^, p40^phox^, and Rac1/Rac2 GTPase) to the plasma membrane where they form a complex with membrane-bound flavocytochrome units, gp91^phox^ and gp22^phox^, in order to form an active nicotinamide adenine dinucleotide phosphate (NADPH) oxidase (Forman and Torres, 2002; Gersuk et al., 2006). In immunosuppressed mice through cyclophosphamide treatment, mice were found to be less susceptible to IA than p47^phox^ deficient mice, which are defective in NADPH ROS generation and a model of CGD. In addition, mice transgenic for p47^phox^ under the control of the human CD68 that allows for targeted NADPH oxidase expression on macrophages and monocytes had increased survival rates compared to the global knockout illustrating the importance of oxidative mechanisms (Grimm et al., 2013). Specifically in AMs, p47^phox^ deficient AMs were unable to control the growth of phagocytosed conidia in contrast to wild-type AMs (Grimm et al., 2013).

AMs play an important role in the inflammatory response through the activation of PRRs and cytokine and chemokine production (Figure 1), which include neutrophil attractants such as macrophage inflammatory protein-2 (MIP-2/CXCL2) and CXCL1(Bhatia et al., 2011). During phagosomal acidification, a phagosome containing conidia fuses with a lysosome in order to form a phagolysosome, which leads to ATPase mediated acidification and activation of enzymes such as chitinases that leads to the degradation of the cell wall exposing ligands for pattern recognition receptors (PRR), TLRs, and Dectin-1 (Ibrahim-Granet et al., 2003; Kasperkovitz et al., 2010; Faro-Trindade et al., 2012).

*In vivo*, clodronate treatment has been used as a method of depletion of AMs (Philippe et al., 2003; Bhatia et al., 2011). Clodronate treated mice were shown to have higher fungal burdens than wild-type mice even though there was an increase in neutrophil recruitment, which can indicate that there is some form of communication between AMs and neutrophils since both seem to be needed in order to control the infection (Bhatia et al., 2011). The mechanism by which this occurs has yet to be fully elucidated and warrants further study. It has been suggested that AMs are able to eliminate low amounts of inocula, and that higher amounts warrant neutrophil activation and recruitment (Philippe et al., 2003). These findings are controversial since there is also evidence that AMs are dispensable in *A. fumigatus* infection, which could be attributed to their use of different strains of mice as well as their use of diverse strains of *A. fumigatus* (Mircescu et al., 2009).

### Neutrophils

*A. fumigatus* also produces immunosuppressive toxins such as gliotoxin and fumagillin, which affects neutrophil function by preventing the formation of a functional NADPH oxidase (Tsunawaki et al., 2004; Fallon et al., 2010). In a mutant strain of *A. fumigatus* in which the *gliP* gene is deleted, infected immunosuppressed mice through corticosteroid treatment had an attenuated virulence compared to non-immunosuppressed mice (Sugui et al., 2007). *GliP* catalyzes the first biosynthetic step in the synthesis of gliotoxin, and deletion prevents its synthesis as well as its effect on NADPH oxidase (Sugui et al., 2007). Neutrophils were found to be a primary target for gliotoxin since neutropenic mice did not differ in virulence when infected with the mutant compared to wild-type *A. fumigatus* (Spikes et al., 2008).

During neutrophil degranulation, azurophil granules expel fungicidal hydrolytic enzymes into the phagocytic vacuole (Segal, 2005). There are two predominant types of granules present in neutrophils: azurophil (primary) granules and specific (secondary) granules (Spitznagel, 1990; Segal, 2005). As mentioned, azurophils contain hydrolytic enzymes for killing and digestion of pathogens whereas specific granules serve as sources of replenishment for membrane components as well as limiting free radical reactions (Segal, 2005). Azurophil granules contain myeloperoxidase, cathepsin G, elastase, and proteinase 3 (Segal, 2005). Specific granules contain lactoferrin (binds and sequesters iron and copper), transcobalamin II, neutrophil gelatinase-associated lipocalin, and other membrane-associated proteins including flavocytochrome b_558_ of the NADPH oxidase (Segal and Jones, 1979; Segal, 2005). NADPH oxidase derived ROS has been shown to promote degranulation and activation of these hydrolytic enzymes (Reeves et al., 2002; Feldmesser, 2006). Activation leads to an accumulation of ROS into the endocytic vacuole, which leads to the accumulation of potassium ions in the vacuole to compensate for the anionic charge from ROS (Reeves et al., 2002). The increase in ionic strength triggers the release of the granule proteins (Reeves et al., 2002). The importance of these granules is demonstrated in mice deficient in the serine protease cathepsin G and/or neutrophil elastase that succumb earlier to *Staphylococcal* and *Candida* infections, but are competent in ROS production (Reeves et al., 2002).

Neutrophils have been shown to be essential innate effectors in defense against *A. fumigatus* (Mircescu et al., 2009; Margalit and Kavanagh, 2015). Due to the high mortality rates of neutropenic mice and their inability to control fungal growth and hyphal formation, they are considered to be the most established model of IA (Stephens-Romero et al., 2005; Mircescu et al., 2009). Neutrophils employ various mechanisms in the elimination of *A. fumigatus* germinating spores such as: ROS generation via NADPH oxidase, lactoferrin production, and through the release of antimicrobial proteases by degranulation (Figure 1; Feldmesser, 2006; Sugui et al., 2007).

The importance of oxidative mediated conidiacidal activity by neutrophils is illustrated in patients suffering from CGD (Grimm et al., 2011). These patients possess mutations in p47^phox^, which leads to a defective ROS generation by NADPH oxidase (Grimm et al., 2011). *In vivo*, studies using NADPH oxidase deficient mice as a model for CGD demonstrated delayed recruitment of neutrophils as well as their inability to contain germinating conidia (Bonnett et al., 2006). Histology from the lung tissue samples of the mice showed hyphal structures as well as extensive damage to the lung tissue in contrast to their wild-type counterparts in both C57BL/6 and BALB/C backgrounds (Bonnett et al., 2006). *In vitro*, the addition of hydrogen peroxide and hypochlorous acid to the NADPH oxidase deficient cells prevented germination indicating the importance of ROS (Bonnett et al., 2006).

Neutrophil extracellular traps (NETs) are extracellular structures that are made of chromatin with proteins from neutrophilic granules attached including neutrophil elastase, myeloperoxidase, cathepsin G, lactoferrin, and gelatinase (Brinkmann et al., 2004). Chromatin is described as the backbone of NETs due to the ability of DNases, but not proteases to degrade it. NETs are formed in response to activation via IL-8, lipopolysaccharide (LPS), bacteria, fungi, or activated platelets (Brinkmann et al., 2004; Brinkmann and Zychlinsky, 2007). Once activated, a subset of neutrophils begin a “suicide” program that leads to the their death and NETosis (Brinkmann and Zychlinsky, 2007). NETs require a respiratory burst, which has been experimentally supported by blocking ROS and preventing NET formation through the use of the oxidase inhibitor diphenylene iodonium (DPI; Brinkmann and Zychlinsky, 2007).

CGD patients, who are more susceptible to IA, have neutrophils that are unable to form NETs when stimulated with bacteria or phorbol myristate acetate (PMA; Brinkmann and Zychlinsky, 2007; Fuchs et al., 2007). The importance of NET formation is controversial, because there is also evidence that neutrophil fungicidal activity is NET-independent (Margalit and Kavanagh, 2015). *In vitro*, human bronchoalveolar lavage neutrophils treated with micrococcal nuclease (MNase) in order to degrade chromatin and prevent NET formation were still capable of fungicidal activity, which indicates that killing is NET-independent (Bianchi et al., 2009). This observation was further supported by another group that treated neutrophils with cytochalasin-D in order to block phagocytosis, and found that neutrophil killing was abrogated (Bruns et al., 2010). This indicates that phagocytosis and not NET formation is the primary killing mechanism by neutrophils in response to fungi (Bruns et al., 2010). The mechanisms as to whether a neutrophil undergoes NETosis upon contact or phagocytosis of fungal elements remain unclear, but in regards to killing, antimicrobial peptides have been suggested to be one possible mechanism (Levitz et al., 1986; Bruns et al., 2010).

Human neutrophils have also recently been shown to produce NETs in response to *A. fumigatus* hyphal structures, but to a lesser extent in response to resting and swollen conidia (Bruns et al., 2010). An *A. fumigatus* mutant that lacks the hydrophobin RodA surface layer of swollen and resting conidia was shown to increase NET formation as compared to wild-type conidia (Bruns et al., 2010). This indicates that a NET inducing element is exposed during hyphal formation when the RodA layer is lost (McCormick et al., 2010). The RodA protein shields the conidia, and prevents the activation of an adaptive immune response (Bruns et al., 2010; McCormick et al., 2010). Another NET associated protein is calprotectin, which has been shown to chelate zinc ions, and inhibit growth of *A. fumigatus* (McCormick et al., 2010; Bianchi et al., 2011). Addition of zinc ions to culture medium was able to rescue the growth inhibition. (McCormick et al., 2010) The effect seen was not *A. fumigatus* specific. *In vitro, A. nidulans* growth was inhibited by blocking calprotectin through S100A9 blocking antibodies or in the S100A9 deficient mouse strain (Bianchi et al., 2011).

### Eosinophils and mast cells

In ABPA, there is enhanced eosinophil recruitment along with fungal enzymes that have been shown to contribute to epithelial damage. In contrast, there is evidence that eosinophils possess fungicidal activity due to the antimicrobial proteins present in their granules (Patterson and Strek, 2014). Eosinophils have recently been shown to play a role in defense against *A. fumigatus* (Lilly et al., 2014). Mice deficient in a high-affinity GATA-binding site in the GATA-1 promoter are depleted of eosinophils (not mast cells or platelets) have been shown to have deficiencies in fungal clearance and increase in fungal burden in comparison to their wild-type counterparts (Lilly et al., 2014). In addition, there was impaired production of cytokines and chemokines such as IL-6, IL-17A, GM-CSF, IL-1β, and CXCL1 (Lilly et al., 2014).

Mast cells have been shown to be key mediators of the pathophysiology of asthma (Bradding et al., 2006). They have been shown to localize in the bronchial smooth muscle bundles in patients with severe asthma such as ABPA patients, but not in normal subjects or those with eosinophilic bronchitis (Bradding et al., 2006). Using the RBL-2H3 cell line and bone marrow-derived mast cell cultures to examine mast cell function, *A. fumigatus* hyphae was shown to adhere to mast cells, and induced their degranulation in an IgE- independent manner unlike conidia and immature hyphae (Urb et al., 2009). Degranulation leads to the release of a variety of enzymatic proteins as well as bioactive substances such as histamine and tryptase, which are important in mediating pulmonary inflammation and airway constriction (Bradding et al., 2006). Although exposure to *A.fumigatus* leads to degranulation of mast cells, mast cells cannot inhibit their growth or metabolic activity (Urb et al., 2009).

### Platelets

Several studies have examined the role of platelets in *A. fumigatus* infection since development of IA leads to hyphal invasion of blood vessels, which can cause thrombosis as well as vascular infarction (Lopes Bezerra and Filler, 2004). *In vitro*, it has been shown that human platelets surround and adhere to hyphal structures as well as conidia, but are unable to phagocytose fungal spores (Christin et al., 1998). In contrast, other studies have indicated that it is *A. fumigatus*-derived serine proteases as well as gliotoxin that lead to platelet activation, and is not contact dependent (Speth et al., 2013). Platelet activation is characterized by the expression of CD62P and CD63 on the cell surface, which are released by α-granules and δ- granules (Perkhofer et al., 2008; Rødland et al., 2010). Platelets incubated with fluorescein isothiocyanate-labeled cell walls lead to the loss of hyphal surface proteins and hyphal cell wall integrity as indicated by the loss of fluorescence via microscopy (Rødland et al., 2010). These results indicate a role for platelets in fungal containment by their ability to damage the fungal cell wall upon exposure through the release platelet microbicidal proteins stored in their granules.

### Natural Killer (NK) cells

Natural Killer (NK) cells have been found to have fungicidal activity, but the mechanisms in which this occurs, are poorly understood (Bouzani et al., 2011; Schmidt et al., 2011, 2013). NK cells have been shown to be responsive to germinating but not resting conidia, and their release of perforins *in vitro* correlated with increased fungicidal activity (Schmidt et al., 2011). IFNγ produced by NK cells could also contribute to fungal clearance by preventing germinating conidia from growing into hyphal structures, which suggests that in addition to functioning as an immunoregulatory molecule, IFNγ can function as an antifungal effector against *A. fumigatus* directly (Park et al., 2009; Bouzani et al., 2011). AMs incubated with NK cells *in vitro* have also been shown to be more effective in killing the conidia than when incubated alone or with IFNγ deficient NK cells. (Park et al., 2009) In neutropenic models that have been depleted of NK cells, the mortality rate is doubled in comparison to neutrophil depleted mice with wild-type NK cell function further suggesting a contribution of NK cells to antifungal defense *in vivo* (Morrison et al., 2003). Although NK cells have been found to have antifungal activity, they do not seem to be essential. In our previous work, mice that lack all lymphocytes including NK cells, T cells, B cells, γδ T cells, iNKT cells, and innate lymphocytes (ILCs), RAG^−∕−^ γC^−∕−^, do not develop IA upon infection (Espinosa et al., 2014). These results indicate that these lymphocytes are not required for defense against *A. fumigatus* infection.

### Dendritic cells (DCs)

Dendritic Cells (DCs) have been shown to have multiple roles in response to *A. fumigatus* infection (Bhatia et al., 2011) (Table 1). DCs phagocytose conidia through PRRs including Dectin-1, Dendritic Cell-Specific Intercellular adhesion molecule-3-Grabbing Non-integrin (DC-SIGN), complement receptor 3 (CR3), and FcγRIII (Bozza et al., 2002; Serrano-Gómez et al., 2005; Mezger et al., 2008). DCs also produce proinflammatory cytokines such as TNFα, IL-6, IL-12, IL-1α, and IL-1β in response to *A. fumigatus* (Bozza et al., 2002; Mezger et al., 2008; Morton et al., 2011). Differential cytokine production by DCs is observed when exposed to different forms of the fungus *in vitro* (Bozza et al., 2002). When exposed to conidia and hyphae, TNFα is produced. IL-12 is produced in response to conidia, and IL-4 and IL-10 in response to hyphae (Bozza et al., 2002). There are three major subtypes of DCs in the lung, conventional DCs (cDCs), plasmacytoid DCs (pDCs), and monocyte-derived DCs (moDCs; Margalit and Kavanagh, 2015).

DCs also produce a variety of chemokines that are needed for the recruitment of a variety of innate and adaptive effector cells to aid in fungal elimination (Scapini et al., 2000; Gafa et al., 2007). DCs are recruited to sites of infection by the production of MIP-1α and MIP-1β by neutrophils (Scapini et al., 2000). *In vitro*, infection of human dendritic cells by *A. fumigatus* conidia triggers the secretion of chemokines for neutrophil and Th1 lymphocyte recruitment (Gafa et al., 2007). DCs release increased amounts of CXCL8, which results in neutrophil recruitment (Gafa et al., 2007). *A. fumigatus* infection resulted also in CCL3, CCL4, CXCL10, and CCL20 productions that induce the migration of effector memory Th1 cells (Gafa et al., 2007; Morton et al., 2011). Together these results indicate a dual role for DCs in the innate as well as adaptive immune response against *A. fumigatus* (Margalit and Kavanagh, 2015).

### Plasmacytoid dendritic cells (PDCs)

pDCs are known as Type I IFN producing cells in response to viral stimulation. They comprise about 0.2–0.8% of total peripheral blood mononuclear cells (PBMCs) in humans, and express TLRs 7 and 9 (Colonna et al., 2004). pDCs link the innate and adaptive immune systems by secreting IFNα and TNFα, and by differentiating into mature pDCs with upregulated major histocompatilibility complex (MHC) and costimulatory molecules capable of priming naive T cells (Colonna et al., 2004).

pDCs are a major source of type I IFN, but the role of pDCs as well as type I IFN has not been extensively examined in regards to fungal infections (Ramirez-Ortiz et al., 2011; Margalit and Kavanagh, 2015). Interferon αβ receptor deficient mice (IFNAR^−∕−^) have been shown to have a higher susceptibility to IA in comparison to wild-type mice, which is consistent with the finding that antibody (120G8) mediated depletion of pDCs make mice more vulnerable to *A. fumigatus* infection (Ramirez-Ortiz et al., 2011). When exposed to hyphae of *A. fumigatus*, human pDCs inhibited their growth, but did not kill the fungus (Ramirez-Ortiz et al., 2011). In addition, there are contrasting studies as to whether pDCs are capable of engulfing *A. fumigatus* conidia, which could be attributed to differences in experimental design for pDC isolation such as using CD303 as a marker for positive selection compared to CD123 (Ramirez-Ortiz et al., 2011; Lother et al., 2014). Apoptosis of pDCs is stimulated upon exposure to the release of cytotoxic molecules by *A. fumigatus* such as gliotoxin, and their death results in the release of antifungal effector molecules such as zinc chelators like calprotectin and iron-binding proteins like lactoferrin (Ramirez-Ortiz et al., 2011). Similar to neutrophils, pDCs have also recently been shown to form Dectin-2 mediated extracellular traps or pETs (plasmacytoid extracellular traps), which have been observed to form around *A. fumigatus* hyphae (Loures et al., 2015). Treatment with blocking antibodies against Dectin-2 led to decreased association of pDCs with hyphae in contrast to Dectin-1, which was similar to untreated pDCs exposed to *A. fumigatus* only (Loures et al., 2015). These results suggest that pDCs can recognize *A. fumigatus* via Dectin-2, which results in antifungal activity through pET formation.

### Conventional dendritic cells (CDCş)

cDCs as well as moDCs are typically identified by their high expression of the integrin CD11c and MHC class II. There are two types of cDCs: CD103^+^ cDCs and CD11b^+^ cDCs (Kopf et al., 2015). They can be distinguished from each other by the markers CD207 (present on CD103^+^ cDCs) and MER proto-oncogene tyrosine kinase (MerTK; present on CD11b^+^ cDCs; Kopf et al., 2015). CD11b^+^ cDCs share common markers with moDCs, but can be differentiated by using the marker for the Fc receptor CD64 that is present on moDCs (Kopf et al., 2015). The development of cDCs as well as pDCs is dependent on the FMS-like tyrosine kinase 3 ligand (Flt3L), which is demonstrated by the lack of CD103^+^ DCs in Flt3L deficient mice (Ginhoux et al., 2009; Merad et al., 2013). Basic leucine zipper transcription factor ATF-like 3 (BATF3) is also required for their steady-state generation similar to CD8α DCs (Edelson et al., 2010), but under certain inflammatory conditions such as mycobacterial infection, other members of the BATF3 family have been shown to have compensatory roles (Tussiwand et al., 2012). In an allergic asthma house dust mite model, it was demonstrated that moDCs as well as CD11b^+^ cDCs are important for sensitization, but CD103^+^ DCs are dispensable (Plantinga et al., 2013). They were able to distinguish the contributions of the difference types of DCs by employing the use of Flt3L deficient mice as well as Langerin- diphtheria toxin receptor (DTR) mice (Plantinga et al., 2013). In Langerin-DTR mice, lung CD103^+^ DCs and lymphoid tissue CD8α DCs are eliminated (Plantinga et al., 2013).

In a model of invasive aspergillosis, CD103^+^ DCs were shown to play an important role in shaping a Th17 response through their IL-2 production via nuclear factor of activated T-cells (NFAT) signaling (Zelante et al., 2015). The absence of IL-2 in pulmonary CD103^+^ DCs led to higher IL-17 production in comparison to T cells cultured with IL-2 competent DCs *in vitro* in response to *A. fumigatus* germlings (Zelante et al., 2015). The impact of IL-2 production by DCs was also assessed using mice lacking IL-2 in all tissues or lacking IL-2 in CD4^+^ T cells as well as mice lacking IL-2 specifically in the CD11c^+^ population (CD11c^cre^IL-2^fl∕fl^; Zelante et al., 2015). Mice deficient in IL-2 in all tissues and in mice lacking IL-2 in CD4^+^ T cells had significantly higher fungal burden, but had increased survival in comparison to CD11c^cre^ IL-2^fl∕fl^. CD11c^cre^ IL-2^fl∕fl^ expressed higher levels of IL-17 and IL-23, which led to a fatal hyperinflammatory Th17 response (Zelante et al., 2015). These results indicate that DCs can function as an important regulator of the inflammatory response upon fungal infection.

### CCR2^+^ inflammatory monocytes (CCR2^+^ Mo)

Macrophage and dendritic cell precursors (MDP) in the bone marrow give rise to Ly6C^hi^ monocytes or inflammatory monocytes, which exit the bone marrow in a CC-chemokine receptor 2 (CCR2)- dependent manner in response to infection to the inflamed tissues (Serbina et al., 2008; Geissmann et al., 2010). They represent approximately 2–5% of circulating white blood cells in the bloodstream of a naïve mouse (Shi and Pamer, 2011). The absence of CCR2 leads to deficiencies in monocyte recruitment to the site of infection (Serbina et al., 2008; Shi and Pamer, 2011). CCR2 is also expressed by other cells such as hematopoietic stem cells (HSCs) as well as a subset of NK cells (Si et al., 2010). Once they reach infected tissues, CCR2^+^ Mo can differentiate into inflammatory macrophages or into TNF and iNOS producing DCs (TIP DCs; Serbina et al., 2003; Auffray et al., 2009; Shi and Pamer, 2011). In the absence of inflammation, CCR2^+^Mo can return to the bone marrow or to the spleen, which can function as a reservoir for circulating monocytes (Serbina et al., 2008; Geissmann et al., 2010; Shi and Pamer, 2011). Humans also have a similar counterpart, which is characterized by CCR2^+^ CD14^+^ CD16^−^ expression (Geissmann et al., 2003).

CCR2 has two primary ligands, CC chemokine ligand 2 (CCL2) and CCL7 that have been shown to be important for monocyte recruitment although the mechanism of action has yet to be fully elucidated (Tsou et al., 2007; Shi and Pamer, 2011). In a theoretical model, it was hypothesized that CC-chemokine ligands establish gradients, which guide monocytes to the site of infection by their association with glycosaminoglycans (Proudfoot et al., 2003; Allen et al., 2007). The finding that amino acid substitutions in CCL2 affected monocyte recruitment supported this idea (Proudfoot et al., 2003). Other CC-chemokine ligands, CCL8 and CCL12, have also been shown to bind to CCR2, but did not have a significant role in monocyte trafficking (Tsou et al., 2007). Migration of monocytes is dependent on various integrin and adhesion molecules such as L-selectin (CD62L), P-selectin glycoprotein ligand 1 (PSGL1), lymphocyte function-associated antigen 1 (LFA1; also known as αLβ2 integrin), macrophage receptor 1 (MAC1; also known as integrin αMβ2), platelet endothelial cell adhesion molecule (PECAM1), and very late antigen 4 (VLA4; also known as integrin α4β1; Ley et al., 2007). These molecules are necessary for proper rolling, adhesion, and migration of CCR2^+^ Mo to infected tissues by a variety of different pathogens (Ley et al., 2007). CCR2^+^ Mo have been shown to play important roles in bacterial, viral, protozoan, and fungal infections (Shi and Pamer, 2011).

In the context of *A. fumigatus* infection our previous studies found an essential role for CCR2^+^ monocyte-derived dendritic cells in the activation of fungus-specific CD4^+^ T cells. In these studies, the role of CCR2 was investigated using a depleter mouse strain, in which the CCR2 promoter drives the expression of the simian diphtheria toxin receptor (Hohl et al., 2009). Administration of diphtheria toxin (DT) leads to the transient depletion of monocytes in the blood, bone marrow, and peripheral tissues (Hohl et al., 2009). Depletion of CCR2^+^ Mo before infection leads to decreased transport of fungal spores to the draining lymph nodes, which prevents *A. fumigatus*-specific CD4^+^ T cell priming (Rivera et al., 2011). Depletion of CCR2^+^ moDCs at later stages of infection, leads to a skewing from a Th1 to a Th17 response in the lung, which indicates that recruitment of monocytes has an influential role in shaping the adaptive immune response and are necessary to promote and sustain Th1 responses (Rivera et al., 2011). Studies with other fungal pathogens further support a conserved function for moDCs in shaping fungus-specific CD4^+^ T cell responses (Traynor et al., 2002; Szymczak and Deepe, 2009; Ersland et al., 2010; Szymczak and Deepe, 2010; Wüthrich et al., 2012b).

In addition to their importance in shaping fungus-specific CD4^+^ T cell responses, CCR2^+^ Mo and moDCs are important direct effectors necessary for prevention of IA (Espinosa et al., 2014). In recent studies, we found that sustained depletion of CCR2^+^ Mo results in the rapid development of IA. Our studies suggest that CCR2^+^ Mo and their derivative moDCs are important direct effectors of fungal spore killing. Moreover, they are necessary to sustain an inflammatory milieu in the lung and the antifungal activity of neutrophils (Espinosa et al., 2014; Caffrey et al., 2015). Altogether, these studies indicate that CCR2^+^ Mo and their derivative moDCs are crucial, non-redundant cells in antifungal defense to *A. fumigatus* by acting both as direct innate effectors and by shaping the response of other innate and adaptive immune cells. The importance of monocytes in defense against IA is likely conserved in humans. Patients with monocytopenia are at increased risk for fungal infections (Vinh et al., 2010; Hsu et al., 2011). Moreover, CD14^+^CD16^−^ monocytes isolated from healthy allogeneic hematopoietic stem cell transplantation donors were shown to phagocytose conidia, and inhibit conidial germination while the CD14^+^CD16^+^ subset was able to produce cytokines (Serbina et al., 2009).

## Concluding remarks

The detrimental impact of fungal infections to diverse patient populations across the globe is likely to continue to rise. The interactions of fungi with host innate cells crucially determine the outcome of infection with multiple innate cells subsets contributing essential protective functions. Future therapeutic interventions aimed at boosting innate immunity are likely to provide significant benefit and help improve the current detrimental outcomes associated with invasive aspergillosis and other invasive fungal infections.

## Author contributions

The author confirms being the sole contributor of this work and approved it for publication.

## Funding

Research in the Rivera lab is supported by the National Institutes of Health under awards granted by National Institute of Allergy and Infectious Diseases R01AI114647-01A1 and National Cancer Institute R21CA167238-01A1 to AR, and fellowship F31 AI098408-01A1 to VE. The content of this article is solely the responsibility of the authors and does not necessarily represent the official views of the NIH.

### Conflict of interest statement

The authors declare that the research was conducted in the absence of any commercial or financial relationships that could be construed as a potential conflict of interest.
